# Supplementary material for: Development of an Image Analysis Pipeline to Estimate Sphagnum Colony Density in the Field
Source: Plants (Basel). 2021 Apr 22;10(5):840. doi: 10.3390/plants10050840 (PMC8143480; doi:10.3390/plants10050840)
Supplement: Supplementary file 1 [file plants-10-00840-s001.zip › plants-1137968-supplementary.pdf]

plants

MDPI

Table S1. Equations used to obtain colour channel image representations, Chromatic difference, HSV difference and Green difference.

Constructed Colour Channels	Equation
Chromatic difference	$\left(\frac{\text{RGB blue}+\text{RGB red}}{2}\right) - \text{RGB green}$
HSV difference	$\frac{(\text{HSV H} * 2) + \text{HSV V}}{2} - \text{HSV S}$
Green difference	$\left(\frac{(\text{RGB green} * 2) + \text{RGB blue}}{2}\right) - \text{RGB red}$

Table S2. General ranges of lower threshold values for channel HSV S. Threshold values are mostly dependent on the amount of non-moss material in an image, lighting and the colouration of the *Sphagnum*.

Image Characterisation	Lower Threshold Range	Lower Threshold Mean	Upper Threshold
Pale discoloured *Sphagnum*, bright image	80-140	107	256
Wet saturated, dark image	50-200	157	256
Intermediates	50-180	110	256
		125	256

Table S3. Location and species distribution of the 68 images that have been thresholded in HSV S and annotated in YCrCb Cb.

Location	Species	n Images
Coed y Darren	*S. quinquefarium*	12
Pen y Garn	*S. fallax*	8
	S. inundatum	4
	S. papillosum	11
	S. fallax & S. papillosum (mix)	4
Llyn Pendam	*S. auriculatum*	11
	S. fallax	10
	S. papillosum	8
Total		68

Table S4. Linear model showing the relationship between the automated counts and the manual counts. This relationship was highly significant and there was no significant effect of species.

Model	Estimate	Std. Error	t-Value	p-Value	R^2	Df	AIC	Residual Standard Error
Intercept (*S. quinquefarium*)	69.88	8.33	8.39	1.38E-11***	0.63	58	513.3	12.57
Manual count	0.483	0.064	7.53	3.81E-10***				
S. fallax	-2.55	4.71	-0.542	0.590 ns				
S. papillosum	9.26	5.34	1.73	0.088 ns				
S. inundatum	-10.69	7.91	-1.35	0.182 ns				
S. auriculatum	-9.56	5.68	-1.68	0.098 ns				

Table S5. Log model showing the relationship between the automated counts and the manual counts. This relationship was highly significant and there was no significant effect of species except for *S. auriculatum*.

Model	Estimate	Std. Error	t-Value	p-Value	R^2	Df	AIC	Residual Standard Error
Intercept (*S. quinquefarium*)	-80.47	26.32	-3.06	0.00337**	0.65	58	509.92	12.24
Log Manual count	100.39	12.66	7.93	8.04E-11***				
S. fallax	-2.31	4.59	-0.50	0.616 ns				
S. papillosum	10.29	5.24	1.97	0.054 ns				
S. inundatum	-10.12	7.70	-1.32	0.194 ns				
S. auriculatum	-11.59	5.42	-2.14	0.037*				

Table S6. The mean value per species of the automated count and the manual count and the resulting correction factor calculated from those values. These correction factors can be used to adjust the automated count to match those of human counters.

Species	Mean Automated Count (Grid)	Mean Manual Count (Grid)	Resulting Correction Factor
S. quinquefarium	126.3	117.0	0.93
S. fallax	120.0	109.2	0.91
S. papillosum	115.6	75.5	0.65
S. inundatum	92.0	68.0	0.74
S. auriculatum	100.5	83.2	0.83

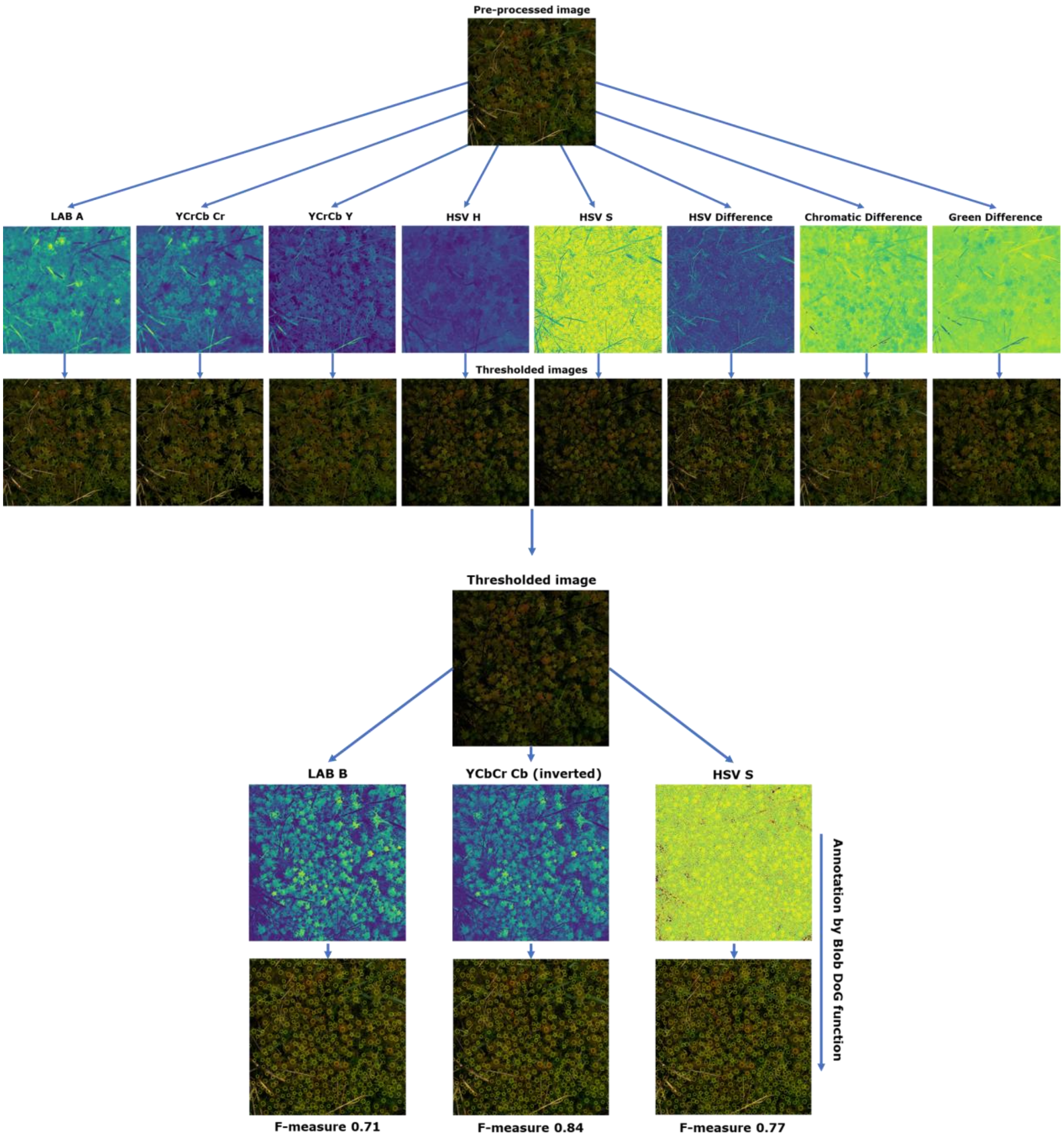

Figure S1. Process workflow visualisation of our method to find the optimal colour channels for thresholding and annotation. An image that has gone through the pre-processing step of the pipeline (Figure 3) was transformed and thresholded in 8 different colour spaces. Subsequently, the 8 thresholded images were transformed again to a different colour space for annotation, using the Blob DoG function from the plantCV package.

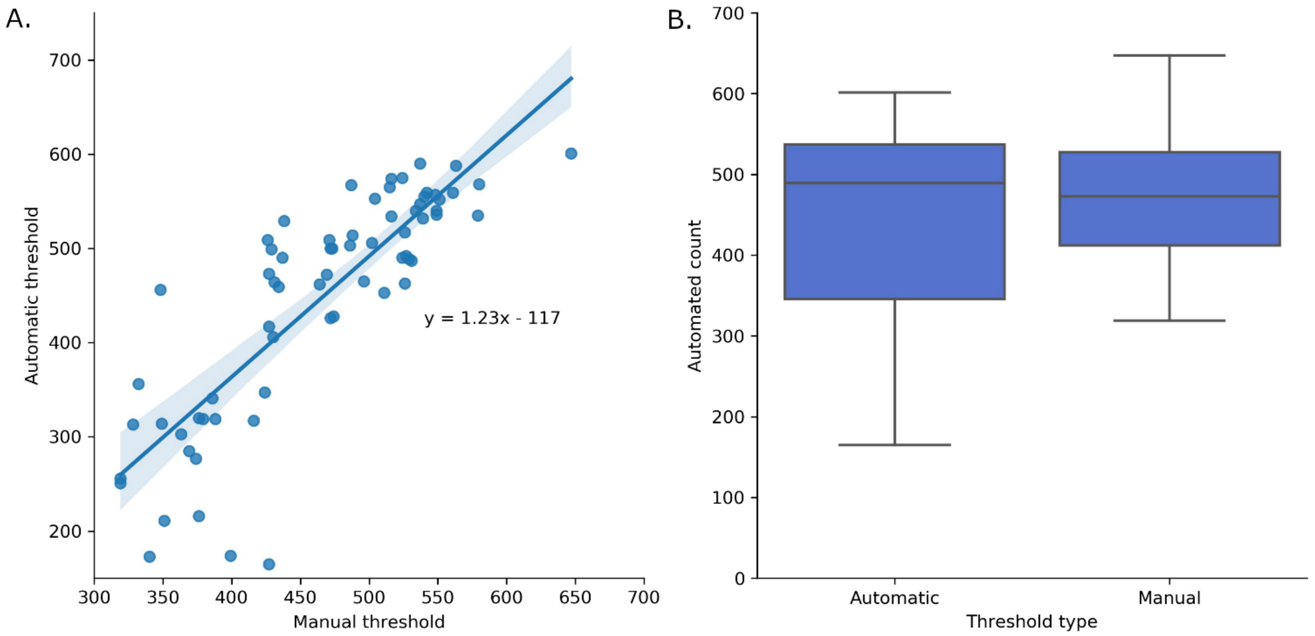

Figure S2. Linear regression (A) of the 68 images (Table S3) and the number of capitula counted after manually thresholding and thresholded using the mean lower threshold value 125 (Table S1). This relationship was highly significant ($p<0.001$***). An ANOVA of the two counts also detected no significant difference (B).

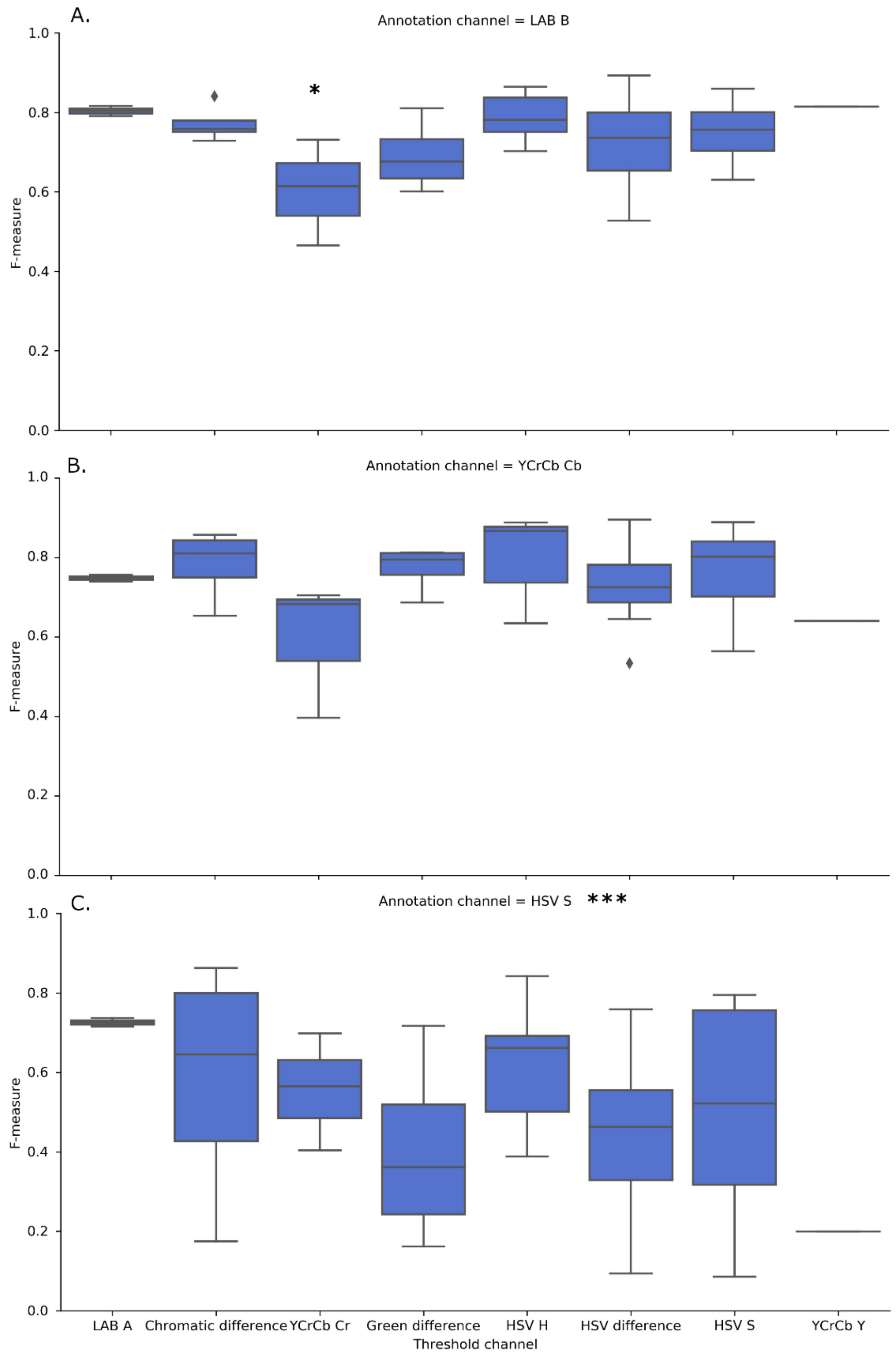

Figure S3. F-measure distributions of the 144 images, annotated the in LAB B (A), YCrCb Cb (B) and HSV S (C) colour spaces (n=48 for each annotation channel, the distribution of the resulting images per thresholding channels were as follows: HSV H (n=6), HSV S (n=15), LAB A (n=2), YCrCb Y (n=1), YCrCb Cr (n=3), Chromatic difference (n=4), HSV difference (n=13) and Green difference (n=4). Asterisks indicate significance calculated using a type III ANOVA (p<0.05*, p<0.001***). Only the thresholding channel YCrCb Cr differed significantly from other channels in performance as quantified by F-measure for annotation channel LAB B. Annotation channel HSV S differed significantly in performance from LAB B and YCrCb Cb.

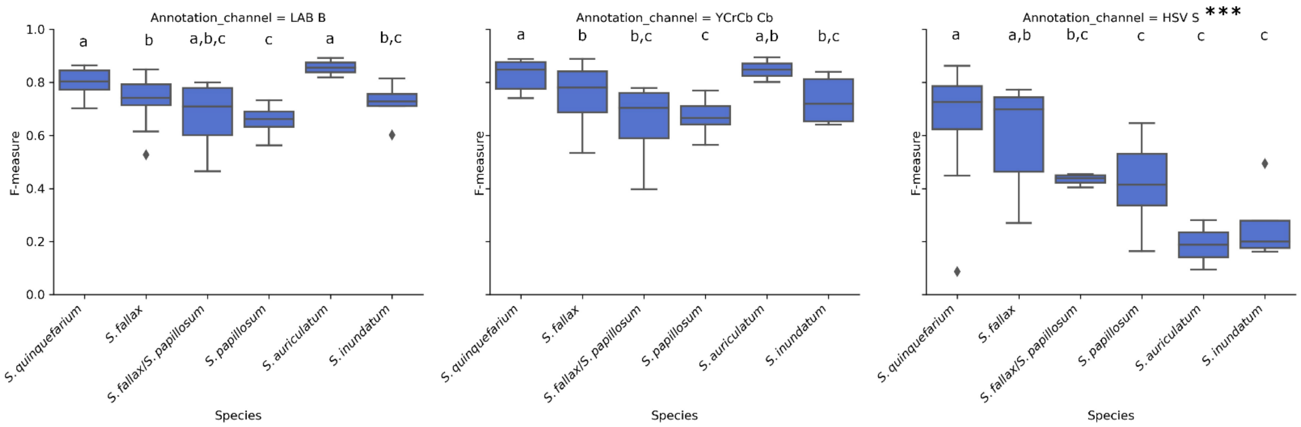

Figure S4. F-measure distributions of the different species for the three annotation channels. Only annotation channel HSV S differed significantly ($p<0.001$), and within the annotation channels the different groups indicate significance ($p<0.05$).
